# Experiences, perceptions, and decision-making capacity towards oral biopsy among dental students and dentists

**DOI:** 10.1038/s41598-023-50323-w

**Published:** 2023-12-22

**Authors:** Juliana Cassol Spanemberg, Rocío Velázquez Cayón, Juliana Romanini, Marco Antonio Trevizani Martins, Pía López-Jornet, Vinicius Coelho Carrard

**Affiliations:** 1 Department of Dentistry, Oral Medicine and Public Health, Faculty of Health Sciences, Universidad Fernando Pessoa Canarias, Gran Canaria, Spain; 2 Department of Dentistry, Oral and Maxillofacial Surgery, Faculty of Health Sciences, Universidad Fernando Pessoa Canarias, Gran Canaria, Spain; 3 Dental Specialty/Oral Medicine Center, Porto Alegre City Hall, Porto Alegre, Brazil; 4 Oral Medicine, Otorhinolaryngology Service, Hospital de Clínicas de Porto Alegre (HCPA), Porto Alegre, Brazil; 5 Oral Medicine, Facultad de Odontología, Universidad de Murcia, Murcia, Spain; 6 School of Dentistry, TelessaúdeRS-UFRGS, Universidade Federal do Rio Grande do Sul, Rua Ramiro Barcelos, 2492/503, Porto Alegre, RS 90035-003 Brazil

**Keywords:** Diseases, Health care

## Abstract

The dentist plays a crucial role in identifying oral lesions as it is their responsibility to conduct the clinical examination for diagnosing diseases in this anatomical region. Dentists should be able to perform simple oral biopsies when this procedure is necessary. However, several studies point out that dentists lack experience and perceive themselves incapable of performing biopsies. This analytical cross-sectional study aimed to assess participants' experiences and perceptions regarding a continuing education activity focused on the biopsy procedure. The secondary aim was to evaluate their ability to determine when a biopsy is indicated. The sample consisted of 228 individuals: 143 dentists and 85 undergraduate dental students who completed questionnaires related to a lecture held in May/2021, as part of the continuing educational activities of the “Red May” Project. Participants completed two questionnaires: the first assessed their experience and self-confidence in performing oral biopsies, whereas the second evaluated their capacity to define when the biopsy is indicated by means the evaluation of 10 clinical cases. The results reveal no significant difference in the percentage of correct answers between dental students and dentists. Regarding the frequency of performing the biopsy procedure, most respondents (69.7%) reported doing so rarely or never. Furthermore, while 31.6% of the participants stated that they perform biopsies depending on the case, 68.4% prefer to refer patients to specialists, such as professionals in Stomatology/Oral Medicine. These findings highlight limitations in the educational preparation of the study population concerning biopsy procedures, oral lesions, and their management. They also indicate a concerning tendency to overestimate knowledge in this area. Thus, this study emphasizes the importance of continuing education and underscores the need to revise academic curricula and provide complementary education for all dental professionals.

## Introduction

The dentist plays a crucial role in identifying oral lesions as it is their responsibility to conduct the clinical examination for diagnosing diseases in this anatomical region^1^. The clinical examination involves gathering general health data and the patient's medical history through anamnesis, as well as objective data through physical examination. This includes examining the oral structures, palpating lymph nodes in the head and neck area, and inspecting the mouth itself^2,3^.

Diagnosing oral diseases relies on recognizing the clinical characteristics of the lesions, often presenting distinct patterns^4^. In many cases, the clinical examination provides enough information to form hypotheses and reach a diagnosis. However, in certain situations, additional tests are necessary for differential diagnosis, especially when distinguishing between similar lesions. The early diagnosis of oral mucosal lesions is based on the subjective evaluation of the operator that could include (or not) the request of complementary tests. Among them, oral biopsy is a crucial procedure in some cases, whereas in others it does not contribute in the diagnostic process^5^. The consequences of not making an accurate diagnosis directly influence the prognosis and life expectancy of patients, mainly in relation to malignances^5–8^.

Biopsy is a procedure that involves the collection of a tissue sample from a living organism for microscopic analysis to establish a definitive diagnosis^9–11^. In essence, biopsy is an invaluable and vital tool for dentists in various scenarios where the clinical examination alone is insufficient to complete the diagnostic process, encompassing benign, potentially malignant, and malignant conditions^12^.

Despite being considered the gold standard in diagnosing oral lesions, dentists often feel uncertain when it comes to performing biopsies^11^. This uncertainty stems from a lack of technical knowledge, inadequate training, and difficulties in interpreting histopathological examination reports. It may also reflect a general limitation in diagnosing oral lesions due to insufficient emphasis on this field of knowledge during undergraduate education^13–15^. According to López-Jornet et al.^16^, dentists should be able to perform simple oral biopsies. However, several studies point out that dentists lack experience and perceive themselves incapable of performing biopsies^14–20^. Apparently, this data is justified by insufficient training or little contact with this type of procedure during graduation^13,15,16^. Recently, it was observed that during or after graduation, less than 25% of dentists perform biopsies^15^ and that only 43% consider themselves capable of performing this procedure^18^. Other factors that could contribute to the low availability to perform the procedure could be the high costs of laboratories and distance from histopathology services^19^. Other concerns related to the procedure include lack of practical ability and the risk of errors in the diagnosis^14^.

Even recognizing that this procedure should be part of their professional routine, the lack of self-confident determine the preference of most dentist to refer patients to specialists^15^. On the other hand, dentists believe that with more training during and after graduation, professional improvement, and performance in carrying out biopsy procedures could be achieved^15^. This approach has its usefulness corroborated by a study funded by the of Regional Government Northwest Spain in which up to 50% of general dentists started to perform biopsies to obtain their diagnoses after an educational intervention^21^. In view of these limitations, it is necessary to understand the reasons that lead dentists to feel insecure or even incapable of performing biopsies and to establish possible ways to mitigate this problem. Therefore, the present study aimed to evaluate experiences and perceptions of dental students and dentists regarding to the oral biopsy procedure. The secondary aim was to assess their ability to determine when an oral biopsy should be indicated.

## Materials and methods

### Study design and ethical considerations

This cross-sectional study was conducted during an educational intervention offered by a collaborative action between the Federal University of Rio Grande do Sul and TelessaúdeRS/UFRGS, a program engaged in actions to support the public healthcare system. The study protocol was approved by the Research Ethics Committee of the Hospital de Clínicas de Porto Alegre (Registry Code: 2021-0627). The data used and/or analyzed during the current study are included in this published article or available from the corresponding author on reasonable request. Informed consent was obtained from all participants. This research was performed in accordance with the Declaration of Helsinki.

### Recruitment of the participants

Primary care dentists were invited to participate of the educational intervention via a disclosure on the TelessaúdeRS/UFRGS’s FacebookTM homepage, Instragram profile, and by e-mail using its mailing list. This intervention was part of the continuing educational activities of the Red May Project (Projeto Maio Vermelho). Red May is an awareness campaign about oral cancer prevention. The campaign alludes to May 31, established by State Law 12.535/06 as the Day to Fight Oral Cancer in the state of Rio Grande do Sul, Brazil. It is a milestone for the population to be aware of signs of lesions in the oral cavity and for health professionals to expand discussions on diagnosis and treatment of the disease.

### Inclusion/exclusion criteria

Initially, all the participants who answered the applied questionnaires were included in the study sample. Then, those who were not dentists or undergraduate dental students, participants with missing data (age, gender, professional category), or those who completed the questionnaire but chose not to participate to the study were excluded.

### Educational intervention

The educational activity consisted of an interview between an undergraduate student and a senior professor and was broadcast in the YouTube Channel of TelessaúdeRS/UFRGS (https://www.youtube.com/@TelessaudeRS) on May 26, 2021. The interview lasted about 48 min and the chat was available to promote interaction with the participants along the activity. It was based on a structured script and its syllabus included topics such as biopsy concept, types, indications, and a brief explanation about the procedure’s steps.

### Evaluation strategy

In the moment of enrollment for the activity, participants were invited to answer two questionnaires. The first addressed questions about demographics, self-confidence and issues that would prevent them from performing a biopsy. Then, a second questionnaire consisted of a test that asked what the decision making would be adopted in front of clinical cases simulated from clinical photographs (pre-test). The photos showed only the oral mucosal lesions and were provided by the professor responsible for the study and did not allow the patients’ identification. The set of cases included fibroma (n = 1), atrophic glossitis (n = 1), geographic tongue (n = 1), traumatic ulcer (n = 1), mucocele (n = 1), leukoplakia (n = 1), secondary syphilis (n = 1), actinic cheilitis (n = 1), squamous cell carcinoma—early (n = 1), and squamous cell carcinoma—advanced (n = 1). The same test was repeated after the educational activity (post-test). All questionnaires were applied via Moodle platform. Participation was voluntary, and the responders were not identified.

### Statistical analysis

Data collected from the Socrative and Google Forms questionnaires were transferred to a database created in Microsoft Excel (Microsoft Corporation, Albuquerque, USA). A descriptive analysis was generated to describe the characteristics of the sample and the answers. Dental students and dentists’ answers were compared by means Mann–Whitney test, since data did not follow a normal distribution, which was verified using the Shapiro–Wilk test. Statistical analyses were performed using SPSS software—version 25.0. The significance level was set at 5%.

### Ethical approval

The study protocol was approved by the Research Ethics Committee of the Hospital de Clínicas de Porto Alegre (Registry Code: 2021–0627).

## Results

The administration of the questionnaire resulted in the participation of a total of 228 individuals, comprising 85 undergraduate students and 143 dentists. Detailed information regarding the participants' demographic data can be found in Table 1.

**Table 1:** Demographics of the study sample.

Variable	
Age	
Mean (SD)	33.2 (11.6)
Min–Max	18–73
Sex, n (%)	
Male	29 (12.7)
Female	199 (87.3)
Professional training, n (%)	
Dental student	85 (37.3)
Dentists	143
General practioner	62 (27.2)
Specialist	68 (29.8)
M.Sc./Ph.D	13 (5.7)
Type of Dental School, n (%)	
Public	132 (57.9)
Private	96 (42.1)
Years since graduation	
Mean (SD)	12.8 (10.6)
Min–Max	0–43
Performance sector, n (%)	
Public	96 (42.1)
Private	16 (7.0)
Both	31 (13.6)
Undergraduate student	85 (37.3)
Region, n (%)	
South	113 (49.6)
Southest	57 (25.0)
Midle-West	4 (1.8)
Northest	41 (18.0)
North	13 (5.7)

Upon inquiring about the frequency with which they engage in the procedure, the responses indicated that 7.9% (n = 18) never perform it, 61.8% (n = 141) rarely do so, 21.9% (n = 50) perform it often, and 8.3% (n = 19) always or almost always carry out the procedure. When exploring decision-making processes in scenarios where a biopsy is necessary, it was revealed that 31.6% (n = 72) of the respondents stated that they perform the procedure depending on the specific case. On the other hand, the remaining participants (68.4%, n = 156) expressed their preference for referring the patient to another healthcare professional (as indicated in Table 2). Among these participants, the most frequently mentioned professional was the specialist in Stomatology/Oral Medicine, accounting for 40.8% (n = 93) of the responses. Table 3 illustrates the opinions of the respondents concerning the reasons for not performing a biopsy on oral lesions. In general, dentists demonstrated higher scores when it came to perceiving difficulties. Both dental students and dentists identified the possibility of diagnostic errors and technical issues as the primary reasons for not conducting the procedure.

**Table 2:** Self-efficacy and perceptions of the participants in relation to oral biopsy (strongly disagree = 1; strongly agree = 5).

	Dental students		Dentists		P*
	Mean (SD)	Median (P25-P75)	Mean (SD)	Median (P25-P75)	
I felt confident to perform biopsy of oral lesions	3.0 (1.0)	3 (2–4)	3.0 (1.1)	3 (2–4)	0.83
I am capable to perform biopsy of soft tissue lesions	3.1 (1.2)	3 (2–4)	2.9 (1.5)	3 (2–4)	0.58
I am capable to perform biopsy of intrabone lesions	2.9 (0.8)	2 (2–3)	2.5 (1.1)	1.5 (1–3)	< 0.01
Biopsy should be performed by all dentists	3..9 (1.2)	4 (4–5)	4.2 (1.4)	5 (4–5)	< 0.01
Biopsy should be performed only by specialists**	2.9 (1.1)	3 (2–4)	2.9 (1.2)	3 (2–4)	0.96

*Mann–Whitney, **Oral Medicine and Oral and Maxillofacial Surgery.

**Table 3:** Opinions of respondents about reason for not performing biopsy of oral lesions (1 = little relevant, strongly relevant = 10).

	Dental students	Dentists	P*
Definition when the procedure is recommended			
Mean (SD)	5.1 (2.7)	5.5 (3.0)	0.27
Median (P25–P75)	3 (1–5)	3 (1–5)	
Technical issues			
Mean (SD)	6.1 (2.3)	7.4 (2.7)	< 0.01
Median (P25–P75)	5 (5–8)	8 (5–10)	
Definition of area to obtain the specimen			
Mean (SD)	5.3 (2.4)	6.3 (2.8)	< 0.01
Median (P25–P75)	5 (4–7)	6 (4–9)	
Definition of amount of tissue to remove			
Mean (SD)	6.0 (2.7)	7.0 (2.8)	< 0.01
Median (P25–P75)	6 (5–8)	8 (5–9)	
Possibility of diagnostic errors			
Mean (SD)	6.3 (2.5)	7.4 (2.7)	< 0.01
Median (P25–P75)	7 (5–8)	8 (5–10)	
Selection of the surgical instruments			
Mean (SD)	4.4 (2.4)	5.4 (3.0)	0.02
Median (P25–P75)	5 (2–6)	5 (3–8)	
Speciment fixation and transport			
Mean (SD)	4.5 (2.9)	6.4 (3.3)	< 0.01
Median (P25–P75)	5 (2–7)	7 (3–10)	
Laboratory working flow			
Mean (SD)	4.7 (2.9)	6.5 (3.3)	< 0.01
Median (P25–P75)	5 (2–7)	7 (4–10)	
Interpretation of histopathological report			
Mean (SD)	5.3 (2.8)	5.5 (3.0)	0.63
Median (P25–P75)	5 (3–7)	5 (3–8)	

*Mann–Whitney.

No notable disparity was observed in the percentage of correct responses between dental students and dentists (Mann–Whitney, p = 0.23), as depicted in Table 4. However, in terms of clinical cases, a statistically significant difference (p < 0.01) was found in only one instance, where the responses of both groups diverged. Roughly 50% of the dentists provided accurate answers regarding the recommended decision-making approach for cases of secondary syphilis, while only 13% of the undergraduate students made the same selection (Table 4).

**Table 4:** Analysis of decision-making capacity in the face of simulated clinical cases (correct responses are highlighted in bold).

	Follow up	Parcial biopsy	Total biopsy	Refer to a specialist	Lab tests	Aspirative punction	P*
Fibroma							
Dental students	8.1	8.1	**56.8**	27.0	0.0	0.0	0.83
Dentists	7.4	5.6	**61.1**	25.9	0.0	0.0	
Mucocele							
Dental students	0.0	10.8	**48.6**	18.9	21.6	0.0	0.14
Dentists	0.0	0.0	**64.8**	18.5	16.7	0.0	
Athrophic glossitis							
Dental students	51.4	8.1	0.0	27.0	**10.8**	2.7	0.55
Dentists	70.4	1.9	0.0	11.1	**16.7**	0.0	
Traumatic ulcer**							
Dental students	**13.5**	24.3	8.1	51.4	2.7	0.0	0.53
Dentists	**18.5**	25.9	1.9	48.1	5.6	0.0	
Geographic tongue							
Dental students	**5.4**	40.5	0.0	48.6	5.4	0.0	0.04
Dentists	**20.4**	24.1	0.0	42.6	13.0	0.0	
Secondary syphilis							
Dental students	8.1	18.9	2.7	54.1	**13.5**	2.7	< 0.01
Dentists	9.3	9.3	1.9	37.0	**42.6**	0.0	
Actinic cheilitis							
Dental students	27.0	**13.5**	2.7	37.8	16.2	2.7	0.30
Dentists	40.7	**22.2**	1.9	27.8	7.4	0.0	
Leukoplakia							
Dental students	5.4	**48.6**	2.7	40.5	2.7	0.0	0.46
Dentists	7.4	**40.7**	3.7	46.3	1.9	0.0	
SCC-early							
Dental students	18.9	**29.7**	10.8	29.7	8.1	2.7	0.72
Dentists	18.5	**33.3**	1.9	38.9	7.4	0.0	
SCC-advanced							
Dental students	0.0	**45.9**	8.1	43.2	2.7	0.0	0.40
Dentists	1.9	**37.0**	1.9	57.4	1.9	0.0	

SCC, squamous cell carcinoma.

*Qui-square test, p < 0.05.

**In this case, the option. “follow-up” was replaced by “extraction and follow-up”.

## Discussion

This cross-sectional study aimed to assess the self-confidence, perceptions, and decision-making abilities of 228 participants who participated in a continuing education program focused on the oral biopsy procedure. After conducting an extensive search of the Brazilian literature, no similar studies were found that explored the attitudes of dentists and undergraduate students towards oral biopsy as a diagnostic tool in oral medicine. While there are numerous studies on oral cancer knowledge and willingness to perform oral biopsies, none specifically target dental practitioners in Brazil.

The literature consistently reports a lack of knowledge about oral diseases, particularly oral squamous cell carcinoma (OSCC), among dental students and dentists from various countries. It has been observed that oral cancer lesions are more frequently detected at an early stage during dental consultations compared to medical check-ups^16,22,23^. A study conducted in Italy found a significant overestimation of knowledge and practice among dentists when comparing their actual and perceived levels of expertise. Nearly half of the participants overestimated their knowledge, and 21.8% overestimated their ability to perform accurate oral examinations. These findings align with the results of the present study and are consistent with the findings of another study^24^. The latter study demonstrated no correlation between individuals' actual knowledge and their perceived knowledge. In other words, participants who considered themselves more knowledgeable (perceived knowledge) had a higher percentage of incorrect answers (actual knowledge).

Regarding decision-making based on simulated clinical cases, the results of dental students and dentists were quite similar. In cases where both groups made correct decisions, this could be attributed to two factors: first, students have recently acquired knowledge as they receive constant instruction at university; and second, dentists gain more knowledge and self-confidence in handling cases over the years of practice. On the other hand, incorrect decision-making may be explained by teaching deficits due to a lack of dedicated theoretical hours on the subject. Additionally, some universities in Brazil do not include clinical practices in oral medicine in their curricula. Therefore, it is possible that some students face challenges in applying their theoretical knowledge to real cases. Another aspect to consider is that, in the general dentistry scenario, most professionals specialize in specific areas and may be less involved in oral medicine issues. In contrast, a study by Pavão et al.^25^ demonstrated that newly graduated dentists scored 2.1 times higher than senior dentists in a knowledge test, highlighting a statistical difference in the knowledge levels between newly graduated dentists and more experienced professionals. Similar results have been reported in previous studies as well^26,27^.

A case-series study conducted in India by Anandani et al.^26^ reported that 50% of general dental practitioners preferred to refer patients requiring biopsies to specialists or higher centers rather than performing the biopsies themselves. This behavior was attributed to a feeling of inadequate experience and a lack of necessary instruments. The lack of consensus regarding the selection of the appropriate area for obtaining the specimen during a biopsy procedure and the storage of surgically removed specimens was also observed in their findings. These findings align with the results of the current study, highlighting the need for more practical training to improve the perception of preparedness for selecting the correct procedure.

A study carried out in Spain by López-Jornet et al.^16^ found that 32.1% of the professionals interviewed resorted to oral biopsy as a diagnostic method, but most of them preferred to refer the patient to other qualified professionals or centers for this purpose, which is consistent with the present series. The researchers emphasized a significant problem identified in their study: some professionals in routine practice do not subject tissue samples to histopathological study. They stressed the importance of dentists being aware not only of how, when, and where to perform a biopsy but also of when to refer the patient to a specialized center, in line with other authors' perspectives^1,25,28,29^.

Carrard and van der Waal^1^ commented that the use of auxiliary tests such as biopsy by general dentists is only justified when professionals are properly trained to perform them. Considering the results of this study, where most participants would refer their cases to independent specialists regardless of their level of education, it indicates a lack of self-confidence in their abilities and knowledge. These findings support the need for increased training hours in universities and/or continuing education courses. Such training programs are particularly beneficial for professionals working in remote areas, as is the case in some countries like Brazil. In Spain, professionals who had participated in continuing education courses were almost four times more likely to perform biopsies on suspicious lesions^30,31^, demonstrating the positive impact of professional updates. Despite the study's limitations, the results suggest a clear need to include oral biopsy clinical skills workshops as a supplementary educational resource for the continuing education of all dental professionals.

According to the results of Mazur et al.^5^, further studies are needed to explore the role of technique-based image analysis to identify a non-invasive early detection method. There are very promising results with artificial intelligence already being applied in the medical area^32^. However, to date, there is no technique based on the evaluation of oral images that can replace biopsy, which remains the gold standard in the diagnosis of malignant lesions.

## Limitations

It is important to acknowledge that the results obtained through the application of questionnaires have certain limitations^19,23,25,33^. In this study, participants' responses may have been influenced by the context of participating in a continuing education course for an oral squamous cell carcinoma (OSCC) prevention campaign. As attendees of such courses are generally more informed about the subject, the findings generated may be overestimated and may not reflect the actual clinical practice of general dentists. Another limitation of this study is that we did not assess the dentists based on their specific specialties within the field of dentistry. This could have affected the variation in answers, as different specialties have varying levels of involvement in surgical procedures.

## Conclusion

The findings of this study highlight the limitations in the educational preparation of the study population concerning biopsy procedures, oral lesions, and their management. These results underscore the crucial role of continuing education initiatives and the need for changes in academic curricula in response to the current global scenario of oral cancer. It is essential to address these limitations and incorporate comprehensive and specialized training in oral biopsy procedures into dental education programs to ensure that dental professionals are adequately prepared to meet the challenges posed by oral diseases.

## References

[1] Carrard VC, van der Waal I (2021). The role of the dentist in the diagnosis and management of patients with oral mucosal diseases. Med. Oral Patol. Oral Cir. Bucal..

[2] López-Jornet P, Camacho-Alonso F, Molina-Miñano F (2010). Knowledge and attitudes about oral cancer among dentists in Spain. J. Clin. Pract.

[3] Pinheiro SMS, Cardoso JP, Prado FO (2010). Knowledge and diagnosis of oral cancer among dental professionals in Jequié, Bahia. Rev. Bras. Cancer.

[4] Leôncio LL, Batista EP, Queiroz FS, Nóbrega CB, Costa LE (2015). Diagnosis and referral of patients with oral diseases in public health services of Patos, Pernambuco, Brazil: Role of the dentist in references and counter-references. Arq. Odontol..

[5] Mazur M, Ndokaj A, Venugopal DC, Roberto M, Albu C, Jedliński M, Tomao S, Vozza I, Trybek G, Ottolenghi L, Guerra F (2021). In vivo imaging-based techniques for early diagnosis of oral potentially malignant disorders-systematic review and meta-analysis. Int. J. Environ. Res. Public Health.

[6] Brocklehurst P, Kujan O, O’Malley LA, Ogden G, Shepherd S, Glenny AM (2013). Screening programmes for the early detection and prevention of oral cancer. Cochrane Database Syst. Rev..

[7] Warnakulasuriya S, Kerr AR (2021). Oral cancer screening: Past, present, and future. J. Dent. Res..

[8] González-Moles MÁ, Aguilar-Ruiz M, Ramos-García P (2022). Challenges in the early diagnosis of oral cancer, evidence gaps and strategies for improvement: A scoping review of systematic reviews. Cancers.

[9] Ghoreishi S, Zargaran M, Baghaei F (2020). Survey of pathology reports with no definitive diagnosis in oral lesions: The necessary skills for the clinicians. Heliyon.

[10] Kumaraswamy KL, Vidhya M, Rao PK, Mukunda A (2012). Oral biopsy: Oral pathologist's perspective. J. Cancer Res. Ther..

[11] Zargaran M (2019). Clinicians’ role in the occurrence of oral biopsy artifacts as a potential diagnostic dilemma. Dent. Med. Probl..

[12] Mota-Ramírez A, Silvestre FJ, Simó JM (2007). Oral biopsy in dental practice. Med. Oral. Patol. Oral Cir. Bucal..

[13] Wan A, Savage NW (2010). Biopsy and diagnostic histopathology in dental practice in Brisbane: Usage patterns and perceptions of usefulness. Aust. Dent. J..

[14] Diamanti N, Duxbury AJ, Ariyaratnam S, Macfarlane TV (2002). Attitudes to biopsy procedures in general dental practice. Br. Dent. J..

[15] Strey JR (2022). Oral medicine experience and attitudes toward oral cancer: An evaluation of dentists working in primary health care. J. Cancer Educ..

[16] López Jornet P, Velandrino Nicolás A, Martínez Beneyto Y, Fernández SM (2007). Attitude towards oral biopsy among general dentists in Murcia. Med. Oral Patol. Oral Cir. Bucal..

[17] Braun LW, Martins MAT, Romanini J, Rados PV, Martins MD, Carrard VC (2021). Continuing education activities improve dentists' self-efficacy to manage oral mucosal lesions and oral cancer. Eur. J. Dent. Educ..

[18] Noro LRA, Rodriguez-Landim J, Cavalcante-de-Andrade-Martins M, Castro-Ponciano-Lima Y (2017). The challenge of the approach to oral cancer in primary health care. Cien. Saude Colet..

[19] Akinyamoju AO, Adeyemi BF, Odofin AD, Balogun AO, Akinyamoju CA (2017). Perception and utilization of oral histopathology services by general practice dentist in Southwest Nigeria. Ann. Ib Postgrad. Med..

[20] Sousa FB, Regina-de-Freitas-e-Silva M, Pessoa-Fernandes C, Goberlânio-de-Barros-Silva P, Negreiros-Nunes-Alves AP (2014). Oral cancer from a health promotion perspective: Experience of a diagnosis network in Ceará. Braz. Oral Res..

[21] Seoane J, Warnakulasuriya S, Varela-Centelles P, Esparza G (2006). Dios PD Oral cancer: Experiences and diagnostic abilities elicited by dentists in North-western Spain. Oral Dis..

[22] Lim K, Moles DR, Downer MC, Speight PM (2003). Opportunistic screening for oral cancer and precancer in general dental practice: Results of a demonstration study. Br. Dent. J..

[23] Pentenero M, Chiecchio A, Gandolfo S (2014). Impact of academic and continuing education on oral cancer knowledge, attitude and practice among dentists in North-Western Italy. J. Canc. Educ..

[24] Leao JC, Goes P, Sobrinho CB, Porter S (2005). Knowledge and clinical expertise regarding oral cancer among Brazilian dentists. Int. J. Oral Maxillofac. Surg..

[25] Pavão-Spaulonci G, Salgado-de-Souza R, Gallego-Arias-Pecorari V, Lauria-Dib L (2018). Oral cancer knowledge assessment: Newly graduated versus senior dental clinicians. Int. J. Dent..

[26] Alaizari NA, Al-Maweri SA (2014). Oral cancer: Knowledge, practices, and opinions of dentists in Yemen. Pac. J. Cancer Prev..

[27] Clovis JB, Horowitz AM, Poel DH (2002). Oral and pharyngeal cancer: Knowledge and opinions of dentists in British Columbia and Nova Scotia. J. Can. Dent. Assoc..

[28] Anandani C, Metgud R, Ramesh G, Singh K (2015). Awareness of general dental practitioners about oral screening and biopsy procedures in Udaipur, India. Oral Health Prev. Dent..

[29] Coulthard P, Koron R, Kazakou I, Macfarlane TV (2000). Patterns and appropriateness of referral from general dental practice to specialist oral and maxillofacial surgical services. Br. J. Oral Maxillofac. Surg..

[30] Seoane-Leston J (2010). Knowledge of oral cancer and preventive attitudes of Spanish dentists. Primary effects of a pilot educational intervention. Med. Oral Patol. Oral Cir. Bucal..

[31] Seoane J, Varela-Centelles P, Esparza-Gómez G, Cerero-Lapiedra R, Seoane-Romero JM, Diz P (2013). Simulation for training in oral cancer biopsy: A surgical model and feedback from GDPs. Med. Oral Patol. Oral Cir. Bucal..

[32] Gomes RFT, Schuch LF, Martins MD, Honório EF, de Figueiredo RM, Schmith J, Machado GN, Carrard VC (2023). Use of deep neural networks in the detection and automated classification of lesions using clinical images in ophthalmology, dermatology, and oral medicine-a systematic review. J. Digit Imaging..

[33] Colella G, Gaeta GM, Moscariello A, Angelillo IF (2008). Oral cancer and dentists: Knowledge, attitudes, and practices in Italy. Oral Oncol..

